# Cardiopulmonary ultrasound correlates of pleural effusions in patients with congestive heart failure

**DOI:** 10.1186/s12872-022-02638-1

**Published:** 2022-04-26

**Authors:** Hong Li, Jian Chen, Ping-xiang Hu

**Affiliations:** 1 Department of Ultrasound, The Fourth Clinical Medical College of Guangzhou University of Chinese Medicine (Shenzhen Traditional Chinese Medicine Hospital), No. 1, Fuhua Road, Futian District, Shenzhen, 518033 China; 2 Department of Ultrasound, The First Affiliated Hospital of Shenzhen University (Shenzhen Second People’s Hospital), No. 3002, Sungang West Road, Futian District, Shenzhen, 518035 China

**Keywords:** Ultrasound, Pleural effusion, Heart failure, Echocardiography

## Abstract

**Background:**

Pleural effusions are common in patients with congestive heart failure. However, there is a need to assess systematically the correlation between effusion volume, extravascular lung water and echocardiographic parameters. We used combined cardiopulmonary ultrasound to evaluate the relationship between effusion volume, extravascular lung water, and echocardiographic parameters in patients with congestive heart failure.

**Methods:**

Patients who were hospitalized for congestive heart failure underwent combined cardiopulmonary ultrasound. A semiquantitative score of pleural effusions was derived by pulmonary ultrasound and extravascular lung water was estimated by ultrasound lung comets. The measurements were compared with echocardiographic and clinical results.

**Results:**

Among 168 patients (median age 66 years, 69.6% men), 102 (60.7%) had pleural effusions, 84.3% bilateral, 10.8% right-sided, and 4.9% left-sided. High pleural effusion scores were associated with high ultrasound lung comet scores (*P* < 0.0001). Compared with patients without pleural effusions, patients with pleural effusions were significantly older and had higher systolic pulmonary artery pressure (SPAP), NT-proBNP, New York Heart Association scale, larger left atrium, larger right ventricle, more severe mitral regurgitation, and worse left and right heart function. Adjusted for age, multiple logistic regression analysis showed that SPAP (OR 5.688, *P* = 0.006) and E/A (OR 3.941, *P* = 0.043) were the significant variables and risk factors associated with pleural effusions in heart failure.

**Conclusion:**

For patients with left heart failure, the degree of pleural effusions was associated with pulmonary congestion. Elevated SPAP and E/A were the main risk factors for the formation of pleural effusions in patients with congestive heart failure.

**Supplementary Information:**

The online version contains supplementary material available at 10.1186/s12872-022-02638-1.

## Introduction

Heart failure is a life-threatening disease worldwide [1, 2]. Pleural effusions are a common minor Framingham criterion for heart failure. Ho et al. reported that pleural effusion secondary to heart failure occurred in 18% of 2,388 patients [3]. Roguin et al. described 150 patients with acute pulmonary edema; only pleural effusions were found to be statistically significant for 1-year mortality [4].

Pulmonary congestion at discharge was associated with readmission and mortality in patients with acute decompensated heart failure [5]. Pulmonary ultrasound can detect accurately pulmonary congestion and pleural effusions with noninvasive and semiquantitative standard protocols [6, 7]. In addition, pulmonary ultrasound has higher sensitivity and specificity compared with clinical exam and chest radiography for detecting pleural effusions [8]. But the pathogenesis of pleural effusions in heart failure is multifactorial and poorly characterized. Particularly, more study by ultrasound and echocardiography is needed to assess the activity of extravascular lung water and cardiac structure/function in pathogenesis of pleural effusions. Thus, in this study, we quantified pleural effusions by applying a semiquantitative score, and we determined the prevalence of pleural effusions and the association between pleural effusions and degree of extravascular lung water, echocardiographic findings, and clinical and laboratory parameters of patients with heart failure.

## Materials and methods

### Study protocol

The Ethics Committee of Shanxi Provincial People's Hospital approved this study, and all participants provided written informed consent. We studied patients with congestive heart failure who were admitted to our Internal Medicine-Cardiovascular Department from August 2018 to July 2020. The diagnosis was based on the 2016 ESC Guidelines [9].

**Fig. 1:**
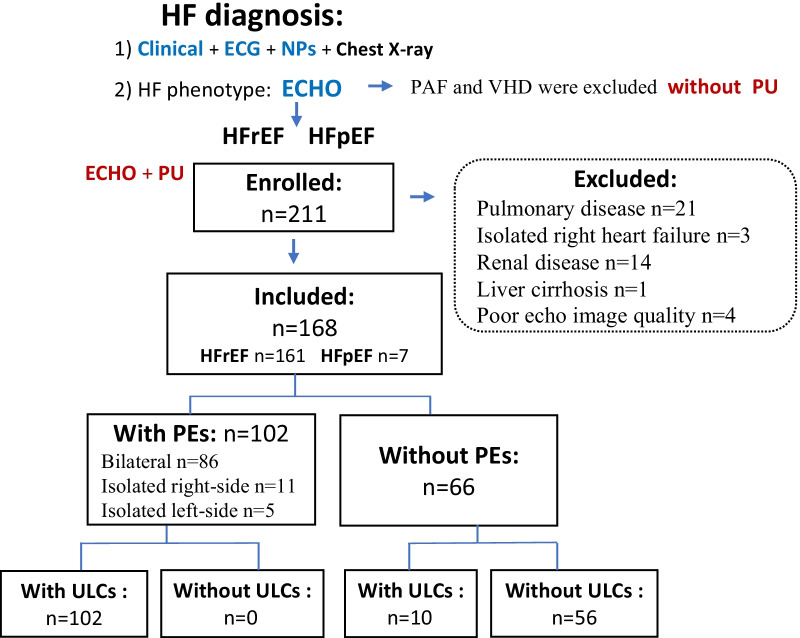
Flowchart of the study. *ECHO* echocardiography; *PU* Pulmonary ultrasonography; *PAF* Persistent atrial fibrillation; *VHD* Valvular heart disease

The following parameters were collected during hospitalization: medical history, body surface area, amino-terminal pro-B-type natriuretic peptides (NT-proBNP), and New York Heart Association (NYHA) functional classification [10]. Patients were excluded if they met one of the following criteria: aged < 18 years, persistent atrial fibrillation, valvular heart disease, isolated right heart failure, systemic disease associated with pleural effusion (such as liver cirrhosis, hypoproteinemia or renal disease), pulmonary disease associated with pleural effusion(pleural line on ultrasound and history or clinical diagnosis to distinguish patients with heart failure-associated pleural effusions from patients with other potential causes) (Fig. 1).

### Cardiopulmonary ultrasonography

The ultrasonographic equipment used for pulmonary ultrasonography and echocardiology was Philips CX50 and Philips APEC 7C (Philips Healthcare, Bothell, Washington, USA) with S5-1 phased-array probe (1-5 MHz). After the transthoracic echocardiography examination, all patients underwent pulmonary ultrasonography with the same phased-array transducer. But when multiple ultrasound lung comets (ULCs) were observed, we used a L12-3 linear probe (3-12 MHz) to observe the pleural lines (also named lung surface, normal pleural line manifests as smooth and regular hyperechoic linear structure with a linear probe. The abnormal pleural line could also generate ULCs such as from interstitial lung disease [11] or pneumonia [12, 13]; thus, patients were excluded if they had abnormal pleural lines observed by linear probe).

Echocardiography was performed in the left decubitus position, supine, or near-to-supine position. Pulmonary ultrasound was examined with patients in supine or near-to-supine position, and pleural effusions were estimated with patients in a semi-recumbent position. The patients were not treated with intravenous diuretics before ultrasonography.

### Pulmonary ultrasonography

**Fig. 2:**
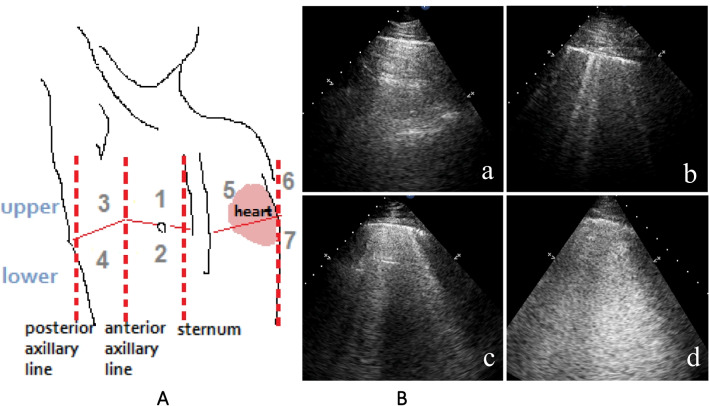
Ultrasound lung comets (ULCs) scoring method. **A** Seven-zones: the anterior zone was delineated from the sternum to the anterior axillary line, and the lateral zone was from the anterior to the posterior axillary line. The anterior and lateral chest walls were subdivided into upper and lower halves with the fourth intercostal space as the boundary from the clavicle to the diaphragm anterior (lower area on the left side was subsequently removed because most of the study population had an enlarged heart which intervened with the area). **B** Increasing severity of interstitial or alveoli involvement. (a) Normal lung = 0: ULCs are absent. (b) Septal syndrome = 1: ULCs are about 7 mm apart, corresponding to subpleural septa. (c) Interstitial-alveolar syndrome = 2: ULCs are confluent. (d) White lung = 3: ULCs have coalesced, resulting in an almost completely white echographic lung field [14]

The ultrasound lung comets (ULCs) scoring method was the following: The anterior and lateral chest wall was divided into seven-zones. ULCs score was then calculated according to the degree of pulmonary edema (Fig. 2). A score of 0 (Zero) defined as absence of ULCs. A score of 1 corresponded to septal syndrome (ULCs at regular distances, corresponding to pleural projection of the subpleural septa which equal to about 7 mm). Interstitial-alveolar syndrome was assigned a score of 2 (ULCs more confluent, separated by < 7 mm). A score of 3 referred to white lung (ULCs were coalesced, resulting in an almost completely white echographic field, confluent B-lines > 80%). The images with the highest score in each zone were recorded. A final ultrasound lung comets score was obtained by summing the ULCs scores of seven-zones of anterolateral chest scans [14].

**Fig. 3:**
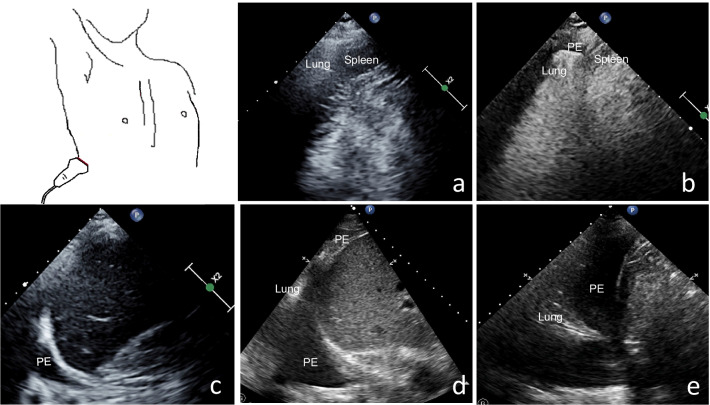
Pleural effusions (PEs) scoring method. **a** No PEs visible = 0. **b** PEs visible only in the costophrenic angle = 1. **c** PEs extending over the costophrenic angle without a clear separation of the lung base from the diaphragm = 2. **d** Clear separation between diaphragm and lung base at any point = 3. **e** PEs occupying more than 50% of the basal pleural cavity visible in the standardized imaging plane = 4 [15]

Pleural effusions (PEs) scoring method: A semiquantitative score from 0 to 4 points was assigned for each hemithorax and 0 to 8 for both thoraces; thus, the total PEs scores were 0 to 8 for each patient [15] (Fig. 3) (Additional files 1–5: Videos 1–5).

Ultrasound lung comets and PEs score were calculated offline by two readers blinded to clinical, laboratory, and echocardiographic data.

### Transthoracic echocardiography

The following data were obtained: Left ventricular end-diastolic diameters (LVEDD), left ventricular end-systolic diameters (LVESD), left ventricular end-diastolic volumes (LVEDV), left ventricular end-systolic volumes (LVESV), left atrial (LA) volume, right ventricle (RV) dimensions are estimated from a RV-focused apical four-chamber view using RV basal diameter, left ventricular ejection fraction (LVEF), tricuspid annular plane systolic excursion (TAPSE), E and A diastolic flow of mitral valve by Pulse Wave Doppler, e′ and a′ diastolic velocity by Tissue Doppler Imaging from the septal and lateral sides of the mitral annulus, the ratio of E to A (E/A) and averaged calculated E/e′ ratio [16]. Mitral regurgitation severity was graded semi-quantitatively by Color Doppler Flow Imaging [17]. The systolic pulmonary artery pressure (SPAP) was estimated by calculating the sum of right atrial pressure and tricuspid valve regurgitation pressure gradient [18].

### Statistical analysis

Data are expressed as mean and standard deviation or percent, and skewed variables as median (interquartile range). Spearman's correlation was used to identify the association between study variables. Pleural effusions and ULCs scores were calculated by a weighted kappa statistic to assess inter- and intra-observer reliability. Multiple logistic regression analysis was performed to identify the significant factors that affect pleural effusion score in heart failure, and collinearity between dependent factors was checked. The Hosmer and Lemeshow Test showed a *p*-value of 0.180, the Nagelkerke R Square value for the model was 0.604, which indicatied that our model fit the data well. Statistical analysis was performed with SPSS 20.0 software (IBM-SPSS Inc., Chicago, IL, USA).

## Results

### Study participants

Table 1 lists baseline characteristics of the participants (n = 168); Fig. 1 shows the flowchart. The median age was 66 (26–91) years, and 69.6% were men. Seven patients had preserved ejection fraction (> 50%), and 47.6% presented with NYHA class III or IV on admission.

**Table 1:** Baseline characteristics of patients with heart failure

Variable	Mean ± SD/N (%)
Age (years)	65.7 ± 14 (26–91)
Men	117 (69.6%)
Body surface area (m^2^)	1.78 ± 0.16
NYHA class III–IV	80 (47.6%)
Pleural effusions	
Bilateral	86 (84.3%)
Right	11 (10.8%)
Left	5 (4.9%)
NT-proBNP (pg/ml)	7641.00 (2439.00–13,241.00)
ULCs	8.00 (4.00–11.00)
Echocardiographic data	
LVEF (%)	36.93 ± 8.38
SPAP (mmHg)	40.40 ± 11.32
LA_index_	41.10 ± 14.29
E/A	1.84 ± 1.16
Average E/e′	18.41 ± 8.06
LV diastolic function	
Grade I	37 (22.0%)
Grade II	75 (44.6%)
Grade III	56 (33.3%)
RV (mm)	34.57 ± 5.14
TAPSE (mm)	18.20 ± 4.15
LVEDD (mm)	59.95 ± 7.14
LVEDV_index_ (ml)	93.90 ± 25.88

*ULCs* ultrasound lung comets; *LVEF* left ventricular ejection fraction; *SPAP* systolic pulmonary artery pressure; *LA*_*index*_ Left atrial volume index; *LV* left ventricular; *RV* right ventricle; *TAPSE* tricuspid annular plane systolic excursion; *LVEDD* Left ventricular end diastolic diameter; *LVEDV*_*index*_ Left ventricular end diastolic volume index

### Pulmonary ultrasonography

Pleural effusions (PEs) were detected in 102 of the 168 patients (60.7%) with the following distribution: bilateral in 86(84.3%), isolated right-sided in 11 (10.8%), and isolated left-sided in 5 (4.9%).

Assessments of PEs and ULCs score were performed for all patients. The median PEs and ULCs scores were 2 (range: 0–6) and 8 (range: 0–20), respectively. The kappa values for the inter- and intra-observer reliabilities of the ULCs score were 0.90 and 0.92, respectively, and the kappa values for the inter- and intra-observer reliabilities of the PEs score were 0.85 and 0.90, respectively.

### Comparison between PEs score, ULCs score, and echocardiographic parameters

Table 1 reports echocardiographic characteristics of the study patients.

There was a significant correlation between the PEs score and each of the following measurements: ULCs score (*r* = 0.673, *P* < 0.001), SPAP (*r* = 0.529, *P* < 0.001), average E/e′ ratio (*r* = 0.421, *P* < 0.001), E/A (*r* = 0.319, *P* < 0.001), severity of mitral regurgitation (*r* = 0.394, *P* < 0.001), Diastolic function classification (*r* = 0.364, *P* < 0.001), LA_index_ (*r* = 0.335, *P* < 0.001), RAP (*r* = 0.346, *P* < 0.001), TAPSE (*r* = 0.221, *P* < 0.05), RV (*r* = 0.225, *P* < 0.05), LVEF (*r* =  − 0.197, *P* < 0.05), age (*r* = 0.292,* P* < 0.01), NYHA class (*r* = 0.516, *P* < 0.001), NT-proBNP (*r* = 0.398, *P* < 0.001). We did not find a significant correlation between PEs score and the following parameters: sex, LVEDD or LVESD, LVEDV, LVESV.

### Parameters between groups with and without pleural effusions

Table 2 shows the baseline and ULCs and echocardiographic values for patients with and without PEs. Compared with patients without PEs, patients with PEs were significantly older, they had higher ULCs scores, SPAP, E/e′, E/A, RAP, NT-proBNP, NYHA class, larger LA, LV, RV, more severe MR, and lower LVEF and TAPSE. Sex and etiology of heart failure did not influence development of PEs. There was also no significant difference between LV size.

**Table 2:** Baseline characteristics of patients with heart failure

Variable	With pleural effusion N = 102	Without pleural effusion N = 66	*P-*value
Age (years)	67.37 ± 14.08	60.58 ± 12.47	0.006
Men (n, %)	69, 67.6%	48, 72.7%	0.484
Etiology of heart failure			
Coronary artery disease	82	46	0.112
Hypertension	5	6	0.452
Dilated cardiomyopathy	8	9	0.224
Myocarditis	3	1	
Autoimmunity cardiomyopathy	1	1	
Noncompaction of ventricular myocardium	0	2	
Alcoholic cardiomyopathy	3	0	
Hyperthyroid cardiopathy	0	1	
Body surface area (m^2^)	1.78 ± 0.18	1.78 ± 0.11	0.95
NYHA class III–IV (n, %)	72, 70.6%	8, 12.1%	< 0.001
NT-proBNP (pg/ml)	9725.00 (3425.00–15,467.00)	1178.50 (682.49–3735.50)	< 0.001
ULCs	9.50 (7.00–11.00)	0.00 (0.00–4.50)	< 0.001
Echocardiographic data			
LVEF (%)	35.62 ± 8.05	40.99 ± 8.16	0.002
SPAP (mmHg)	44.23 ± 9.25	28.85 ± 8.38	< 0.001
LA_index_ (ml/m^2^)	43.68 ± 12.82	33.12 ± 15.78	< 0.001
MR			< 0.001
None	1	11	
Mild	40	47	
Moderate	44	6	
Severe	17	2	
E/A	2.03 ± 1.19	1.26 ± 0.82	< 0.001
Average E/e′	19.90 ± 8.24	13.76 ± 5.52	< 0.001
LV diastolic function			< 0.001
Grade I	10	27	
Grade II	46	29	
Grade III	46	10	
TAPSE (mm)	17.73 ± 4.05	19.61 ± 4.22	0.034
RAP			< 0.001
< 5 mmHg	55	56	
5–10 mmHg	27	9	
10–20 mmHg	20	1	
RV (mm)	35.25 ± 5.28	32.48 ± 4.11	0.003
LVEDD (mm)	59.27 ± 7.11	60.17 ± 7.17	0.534
LVEDV_index_ (ml/m^2^)	71.29 ± 26.09	60.10 ± 20.71	0.871

*ULCs* ultrasound lung comets; *LVEF* left ventricular ejection fraction; *SPAP* systolic pulmonary artery pressure; *LA*_*index*_ Left atrial volume index; *MR* mitral regurgitation; *LV* left ventricular; *TAPSE* tricuspid annular plane systolic excursion; *RAP* right atrial pressure; *RV* right ventricle; *LVEDD* Left ventricular end diastolic diameter; *LVEDV*_*index*_ Left ventricular end diastolic volume index

Multiple logistic regression analysis adjusted for age showed that elevated SPAP (OR 5.688, *P* = 0.006) and E/A (OR 3.941, *P* = 0.043) were the significant variables associated with PEs in heart failure (Table 3).

**Table 3:** Multiple logistic regression analysis of echocardiographic variables

Variable	Odds ratio	B	S. E	*P-*value	95% CI OR	
					Lower	Upper
SPAP	5.688	0.178	0.064	0.006	1.054	1.355
E/A	3.941	1.179	0.594	0.043	1.015	10.415
LVEF	2.137	− 0.086	0.059	0.144	0.817	1.030
LA_index_	0.150	− 0.013	0.035	0.699	0.922	1.056
Average E/e′	1.025	− 0.058	0.057	0.311	0.843	1.056
MR	0.592	0.533	0.692	0.442	0.439	6.621
TAPSE	0.644	− 0.092	0.115	0.422	0.728	1.142
RV	0.280	− 0.051	0.096	0.597	0.787	1.148
RAP	1.489	− 0.876	0.718	0.222	0.102	1.701

*LVEF* left ventricular ejection fraction; *SPAP* systolic pulmonary artery pressure; *LA*_*index*_ Left atrial volume index; *LV* left ventricular; *MR* mitral regurgitation; *TAPSE* tricuspid annular plane systolic excursion; *RV* right ventricle; *RAP* right atrial pressure

## Discussion

Pleural effusions are often caused by malignancy, congestive heart failure, pneumonia, tuberculosis, rheumatoid disease, cirrhosis, lupus erythematosus, and chylothorax. Pulmonary ultrasound can diagnose some lung diseases in patients with dyspnea often with higher sensitivity and specificity than chest X-ray and without radiation, especially for the detection of pleural fluid [19–21]. The prevalence of PEs depends on the imaging modality. In our study, 102 of 168(60.7%)patients had PEs, a greater prevalence than the (50%) prevalence reported by Morales-Rull, et al. for PEs assessed by chest X-ray of patients with acute decompensated heart failure [22], higher prevalence of PEs by pulmonary ultrasonography should result from chest X-ray is not sensitive in revealing small amounts of fluid, the other explanations including technics, patient’s position, and radiolucent material [23]. In addition, we found that the prevalence of right-sided PEs was greater in patients with heart failure compared with left-sided PEs (11 vs 5 patients). This right-side dominance was reported by other investigators [24, 25]. A higher prevalence of right-sided PEs was explained by the fact that patients with heart failure prefer the right lateral decubitus over supine or left decubitus position, and they spend more time in the right lateral position to avoid discomfort (such as dyspnea) related to the elevated left ventricular filling pressure, larger left ventricle, and reduced cardiac output [25, 26]. However, no mechanism is universally accepted or proven experimentally.

We used cardiopulmonary ultrasound to measure PEs, ULCs, echocardiographic, and clinical characteristics of patients with heart failure. Cardiopulmonary ultrasound minimized the amount of time required to assess the features. The degree of pulmonary edema (expressed by ULCs) correlated well with the PEs score in patients with heart failure. This correlation may be due to the following mechanisms: with a decrease of left ventricular diastolic function, increased left ventricular filling pressure leads to an increase in left atrial pressure. Pulmonary venous hypertension could increase capillary pressure by increasing the back pressure in these veins and could then increase the fluid filtration from the capillaries. Pulmonary edema fluid traverses the visceral pleura and enters the pleural space as a filtrate of interstitial and alveolar edema, so the volume of PEs correlates well with the degree of ULCs. Experimental studies showed that PEs develops when extravascular lung water has reached a certain level in a certain amount of time [27]. When the pulmonary interstitium, alveoli or pleural cavity are filled with water, ultrasound can detect and measure the degree of pulmonary edema and PEs semi-quantitatively in real time and noninvasively.

In this study, PEs score correlated with left ventricular diastolic and systolic function parameters and right ventricular function parameters. In addition, we also found that, comparted with patients who did not have PEs, patients with PEs were significantly older and showed more depressed LV diastolic and systolic function, higher SPAP, a larger left atrium and right ventricle, more severe mitral regurgitation, and worse right ventricular function. Multiple logistic regression analysis showed that SPAP and E/A ratio were the echocardiographic predictors of PEs development, which is consistent with the finding that an elevated E/A ratio was a risk factor for the formation of heart failure-related PEs following the development of severely decreased left ventricular diastolic function [28]. Johns et al. showed that increased RV dimension and decreased left atrial function dimensions are associated with PEs in cats with heart failure [29]. Wiener-Kronish et al. found that the mean pulmonary artery wedge pressure was higher in patients with PEs compared with patients who did not have PEs [30]. Left ventricular dysfunction may lead to increased left atrial pressure, backward pressure transmission and passive increase of PASP. Ultrasound lung comets are affected mainly by left ventricular filling pressure in patients with left heart failure; the good correlation between PEs and ULCs indicated that the influencing factors of PEs may come mainly from the influence of left ventricular filling pressure. In addition, PASP and E/A ratio are important parameters to evaluate left ventricular diastolic function; thus, that the main influencing factor of PEs in patients with left heart failure is left ventricular diastolic function.

Wiener-Kronish et al. reported that chronic pulmonary hypertension alone or accompanied by right atrial hypertension was not associated with the formation of pleural effusions but left-sided heart failure and pulmonary venous hypertension were associated with the formation of transudative PEs in heart failure [30–32]. However, we found that patients with PEs had higher PASP and RAP compared with patients that were without effusions. The difference between our study and previous reports may be due to different study populations; the patients in our study all had left heart failure, but prior research focused on patients with pulmonary arterial hypertension in the intensive care unit and not only on left heart failure. As an important parameter to evaluate left ventricular diastolic function estimated by tricuspid regurgitation [33], elevated PASP in patients with left heart failure reflects the degree of decrease of left ventricular diastolic function. This decrease further causes an increase of RAP and leads to pulmonary congestion and PEs; however, concomitant elevation of RAP is not necessary for the presence of PEs.

In our study, patients with PEs had higher levels of NT-proBNP; the elevation may have resulted from pressure overload of chambers, reduced LVEF, and elevated SPAP, which are associated with greater secretion of natriuretic peptides [34].

### Limitations of this study

This investigation was a small cohort, single-center study. The PEs score was estimated only on the lung base and not the entire field of the lung; however, the accuracy of the method was confirmed by chest computed tomography [15]. The semi-quantitative method of extravascular pulmonary water was estimated by pulmonary ultrasound instead of invasive transpulmonary thermodilution measurements; in our previous studies, ULC score correlated with radiologic score. In our cohort, SPAP was a strong predictor of PEs development; however, we did not have SPAP data for 18 of the 168 patients.

## Conclusion

The PEs score was associated with the degree of the pulmonary congestion in patients with heart failure. Elevated SPAP and E/A were the main risk factors for the formation of PEs in patients with congestive heart failure. The degree of PEs was mainly affected by left ventricular function, especially left ventricular diastolic function.

## Supplementary Information

**Additional file 1**. No PEs visible=0.

**Additional file 2**. PEs visible only in the costophrenic angle=1.

**Additional file 3.**. PEs extending over the costophrenic angle without a clear separation of the lung base from the diaphragm=2.

**Additional file 4.** Clear separation between diaphragm and lung base at any point=3.

**Additional file 5**. PEs occupying more than 50% of the basal pleural cavity visible in the standardized imaging plane=4.
